# Gleanings from Our Exchanges

**Published:** 1874-03

**Authors:** 


APPOINTMENT.—Surgeon-General Barnes, of the United States Army, has been elected a corresponding member of the Academy of Medicine, of France, by a vote of forty-two out of forty-six.—*N. Y. Med. Journal.*

RESUSCITATION FROM CHLOROFORM-NARCOSIS (*The New Orleans Med. and Surg. Jour.*, November, 1873).—In the course of an extended experience in the administration of chloroform, it has happened three times to Dr. M. Schuppert that, to all appearances, the narcotized subject died—that is, respiration ceased, the heart stopped beating, and muscular contractility became extinct. The method he adopted for resuscitating these patients consisted in reversing the body, either by hanging them up by the feet, or laying them over a bed or table, so that the greater part of the body, with the head, hung down. In that position, artificial respiration was also tried. In one case, five minutes elapsed before there was a natural inhalation. All of them recovered. Dr. Schuppert believes that, in cases of death from chloroform, the primary cause of the cessation of the respiration and the circulation, rests in anæmia of the brain, and not in impregnation of the blood with carbonic acid.—*Ph. Med. Times.*

---

ACETIC ACID IN PSORIASIS (*New York Med. Jour.*, January, 1874).—Dr. Buck, of Lubeck, says that by ordinary methods of treating psoriasis, the eruption may be temporarily removed without difficulty; but no protection from relapses is afforded. He believes that in nearly all chronic cutaneous eruptions, and especially in psoriasis, an etiological treatment is impossible, and a permanent result is promised only by a consistent external treatment. He has obtained the best results from the use of acetic acid. After the epidermal growths have been softened and loosened by several warm baths and soap, the glistening scales are removed with a soft brush. At first, a few points of the eruption are to be penciled once a day; and this is to be repeated until the skin remains smooth and feels normal to the touch. There are never any scars left. The treatment lasts from four to six or eight weeks, and, properly carried out, is not followed by relapses.—*Phil. Med. Times.*

THE NEW LOCAL ANÆSTHETIC.—The observation of Horwath, of Kiew, that absolute alcohol at a temperature of 20° Fahr. is a most efficient local anæsthetic, deserves to be remembered and acted upon. He finds it far superior to cold ether, or ice, or the spray of volatile substances.—*Philadelphia Medical and Surgical Reporter*.

